# A vascularized model of the human liver mimics regenerative responses

**DOI:** 10.1073/pnas.2115867119

**Published:** 2022-06-28

**Authors:** Arnav Chhabra, H.-H. Greco Song, Katarzyna A. Grzelak, William J. Polacheck, Heather E. Fleming, Christopher S. Chen, Sangeeta N. Bhatia

**Affiliations:** ^a^Harvard University–MIT Division of Health Sciences and Technology, Massachusetts Institute of Technology, Cambridge, MA 02139;; ^b^Institute for Medical Engineering and Science, Massachusetts Institute of Technology, Cambridge, MA 02139;; ^c^David H. Koch Institute for Integrative Cancer Research, Massachusetts Institute of Technology, Cambridge, MA 02139;; ^d^Biological Design Center, Department of Biomedical Engineering, Boston University, Boston, MA 02215;; ^e^Joint Department of Biomedical Engineering, University of North Carolina at Chapel Hill and North Carolina State University, Chapel Hill, NC 27599;; ^f^Wyss Institute for Biologically Inspired Engineering, Harvard University, Boston, MA 02115;; ^g^Howard Hughes Medical Institute, Massachusetts Institute of Technology, Cambridge, MA 02139

**Keywords:** hepatocyte, regeneration, vascular

## Abstract

Liver regeneration is a well-orchestrated process that is typically studied in animal models. Although previous animal studies have offered many insights into liver regeneration, human biology is less well understood. To this end, we developed a three-dimensional (3D) platform called structurally vascularized hepatic ensembles for analyzing regeneration (SHEAR) to model multiple aspects of human liver regeneration. SHEAR enables control over hemodynamic alterations to mimic those that occur during liver injury and regeneration and supports the administration of biochemical inputs such as cytokines and paracrine interactions with endothelial cells. We found that exposing the endothelium-lined channel to fluid flow led to increased secretion of regeneration-associated factors. Stimulation with relevant cytokines not only amplified the secretory response, but also induced cell-cycle entry of primary human hepatocytes (PHHs) embedded within the device. Further, we identified endothelial-derived mediators that are sufficient to initiate proliferation of PHHs in this context. Collectively, the data presented here underscore the importance of multicellular models that can recapitulate high-level tissue functions and demonstrate that the SHEAR device can be used to discover and validate conditions that promote human liver regeneration.

## Significance

Liver disease causes ∼2 million annual deaths, yet medical treatments and transplantable organs are both lacking. The liver can regenerate when mature hepatocytes divide, and while this process is well studied in rodents, parallel study of human biology has been impossible. We developed a microfluidic device that allows us to manipulate fluid flow, circulating cytokines, and/or paracrine interactions between liver and vascular cells, in order to model multicellular aspects of human liver regeneration. We found that physiologically relevant shear stresses increased the secretion of angiogenesis- and regeneration-associated factors, including prostaglandin E_2_ from endothelial cells, and induced primary human hepatocytes to enter the cell cycle. Next, we can dissect the resulting secretome data to identify factors that stimulate liver regeneration.

The liver possesses a unique capability to return to a constant size within a short time period after tissue loss (1–3). The most common model for studying liver regeneration is the two-thirds partial hepatectomy (PHx), which was first described in rats by Higgins in 1931 (4). During PHx, a large portion of the liver mass is resected, after which a coordinated regenerative response follows. The response involves cytokines, growth factors (5), increases in portal blood flow (6), and a dynamic interplay between hepatocytes (Heps) and nonparenchymal cells (7–9). Liver sinusoidal endothelial cells (LSECs) play a unique role by releasing paracrine-mediated growth factors (8, 10). Nonparenchymal cells are crucial for signal transduction as well as synthesis and secretion of cytokines and growth factors, which play complex regulatory roles in the process of liver regeneration. Various studies have elucidated that the IL-1R signaling pathway plays important roles in liver regeneration after acute liver failure and partial hepatectomy, although the exact mechanisms remain to be established (11). Despite the progress in elucidating which factors, pathways, and cell types participate in liver regeneration, the current model systems are largely based on observations made using rodent cells (12). While such models are plentiful, they often cannot isolate the contributions of the processes listed above, and the exact mechanisms and the interactions between the cellular identities in human liver are largely unknown. Beyond just anatomic differences, such as the presence or absence of lobation, there are also significant variations in ligand-dependent signaling pathways between rodent and human livers (13, 14). Thus, a three-dimensional (3D) model of liver regeneration that allows for paracrine interactions between human hepatocytes and human endothelial cells, and control over physiological inputs such as fluid flow would significantly improve our understanding of the process.

Mechanisms mediating liver regeneration are well studied in rodent models. Using the PHx model in mice, Ding et al. showed that LSECs release angiocrine factors such as Wnt2 and hepatocyte growth factor (HGF), which augment hepatic proliferation (8, 15). Another secreted factor, prostaglandin E_2_ (PGE_2_), was shown to be a master regulator of liver regeneration in zebrafish (16). While these studies focused on soluble factors involved in regeneration, Lorenz et al. attempted to uncover a physiological trigger (17). They showed a correlation between increased blood flow in sinusoids and liver regeneration in mice, but were not in a position to report on how flow-dependent stimuli play a specific role in this process. Furthermore, little is known about how the regenerative process occurs in humans. Although precise media manipulations and coculture configurations have enabled the successful maintenance of human hepatocytes in vitro (18–30), these platforms do not incorporate physiological inputs such as shear stress or paracrine interactions between hepatocytes and endothelial cells that are necessary for modeling liver regeneration. Most microfluidic liver platforms that incorporate fluid flow either do not recapitulate multicellular paracrine interactions (31–33) or do not elicit human hepatocyte proliferation in response to proregenerative stimuli (34).

Here we developed a microfluidic device called structurally vascularized hepatic ensembles for analyzing regeneration (SHEAR) by incorporating multiple design parameters to model the flow-dependent paracrine aspects of human liver regeneration. We first reviewed existing, published literature to identify critical aspects for liver regeneration: 1) hemodynamic alterations such as increased fluid flow, 2) biochemical inputs such as circulating cytokines that are necessary for promoting regeneration, and 3) paracrine interactions between parenchymal and nonparenchymal cells, specifically hepatocytes and endothelial cells. In order to synthesize these features into a bioinspired, functional platform, we fabricated organotypic microfluidic devices with perfusable endothelialized channels that can accommodate fluid flow changes. The lumen of the channel, which functionally represents the sinusoidal capillaries in a human liver, was embedded within an extracellular matrix (ECM) and lined with human endothelial cells. For the parenchymal component, we utilized 3D spheroids composed of primary human Heps (PHHs) and human dermal fibroblasts (HDFs), which our laboratory has previously shown to exhibit in vitro phenotypic hepatic stability in preaggregated constructs over a period of several weeks (35, 36). To mimic key aspects of regeneration, we exposed the multicellular SHEAR device to fluid flow and cytokines via perfusion of the central lumen. By quantifying secreted factors present in the flow through of the device, we delineate the effects of hemodynamic inputs such as shear stress and of biochemical inputs such as cytokines on endothelium- and hepatocyte-derived paracrine factors. Specifically, we show that stimulation by cytokines within fluid flow passing through the central channel promotes cell-cycle entry of human hepatocytes cultured within the device and leads to increased secretion of factors such as PGE_2_. Using PGE_2_ as a candidate regenerative factor, we show that PGE_2_ is endothelium derived and also necessary for cytokine-dependent cell-cycle entry of primary human hepatocytes. Collectively, the data presented here depict the SHEAR device serving as a valuable model for gaining mechanistic insight into liver regeneration by enabling systematic deconstruction of the component inputs and can serve as a platform for discovery of factors that promote human liver regeneration.

## Results

### Vascularized Human Liver Model Supports Hepatocyte and Vascular Function.

**Fig. 1.**
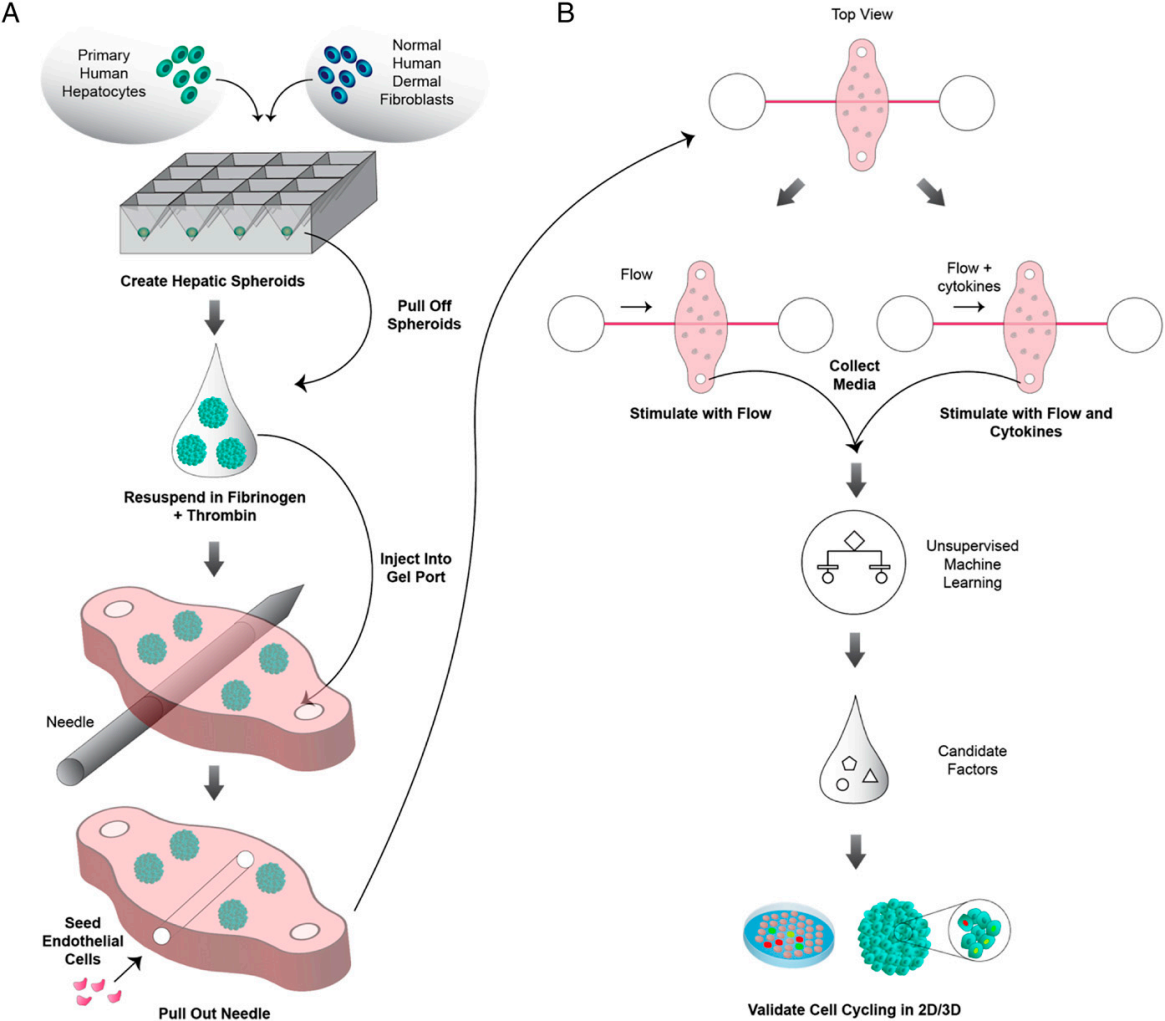
A SHEAR platform models vascularized human liver to uncover flow-dependent paracrine regenerative signals. (*A*) Primary Heps are admixed with normal human dermal fibroblasts (NHDFs) and aggregated in pyramidal microwells to form spheroids. These spheroids are resuspended in fibrinogen and thrombin and polymerized to form a fibrin gel that surrounds a needle. After the needle is retracted, the lumen that is left behind is seeded with primary human endothelial cells, which migrate and form a tight vascular barrier. (*B*) To model liver regeneration on the device, input parameters such as flow and cytokines are modulated. Specifically, in one condition fluid flow is applied through the channel and in the other, a combination of flow and cytokines associated with liver regeneration are applied. The effluent from the various conditions is collected and assayed for secreted factors. Based on unsupervised machine learning, candidate factors are selected and validated to induce Hep proliferation in primary hepatocytes cultured in 2D and 3D configurations.

To create an in vitro model that functionally recapitulates key aspects of human liver regeneration, we mined published accounts to identify the acute changes that happen after a partial hepatectomy. Increases in systemic circulation of cytokines and growth factors, increases in portal blood flow, and increases in paracrine interactions between hepatocytes and nonparenchymal cells, specifically endothelial cells, have all been reported (8, 10, 15). To incorporate these elements into a model platform, we fabricated a microfluidic chip called SHEAR (Fig. 1). SHEAR harbors two linked compartments: a parenchymal compartment that is embedded in a biomaterial through which runs a lumenized endothelial compartment (*SI Appendix*, Fig. S1). In line with our previous findings that HDFs help stabilize the phenotype of PHHs in vitro (35, 36), we utilized admixtures of HDFs and PHHs that we aggregated into spheroids as the cellular component of the parenchymal compartment. While our SHEAR device is modular and amenable to numerous synthetic and natural extracellular matrices within which to embed these spheroids and the lumenized channel, we picked fibrin as the biomaterial of choice due to its innate angiogenic properties (37). Through the center of the spheroid-laden fibrin matrix passes a patent, lumenized, vessel structure that is lined with human umbilical vein endothelial cells (HUVECs), enabling us to perfuse the chip with soluble, media-borne signals. The act of perfusion also imparts fluid pressures that stimulate the endothelial compartment of the system with circumferential stretch and luminal shear stress. Material properties of the fibrin gel also enable the parenchymal and endothelial compartments to interact via diffusion and advection of secreted factors.

**Fig. 2.**
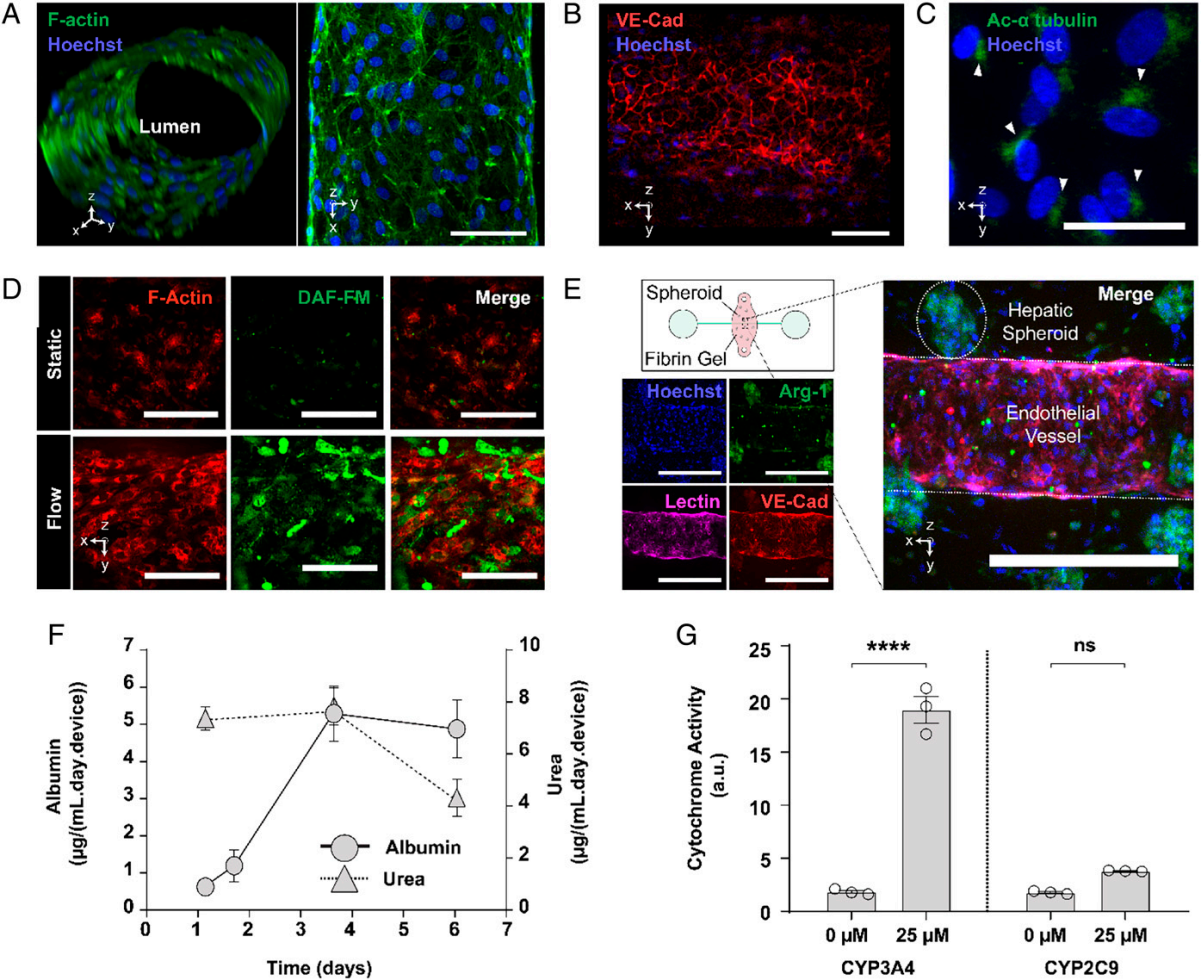
SHEAR devices support functional human hepatocytes and endothelial cells. (*A*) Immunofluorescence analysis of SHEAR devices after 3 d in culture with flow, depicting a patent lumen (confocal 3D rendering on the *Left* and maximum intensity projection on the *Right*. (Scale bar, 100 µm.) Orientation of each image is indicated on the *Bottom Left*. (*B*) Expression of VE-Cad throughout the channel of SHEAR devices, imaged after 3 d in culture with flow (maximum intensity projections). (Scale bar, 100 µm.) (*C*) Expression of primary cilia in SHEAR devices (represented by acetylated α-tubulin), imaged after 3 d in culture with flow (maximum intensity projections). (Scale bar, 100 µm.) (*D*) Immunofluorescence analysis of SHEAR devices after 3 d in culture in the absence and presence of flow, specifically showing F-actin alignment (*Left*) and nitric oxide production (*Center*, DAF-FM, 4-amino-5-methylamino-2′,7′-difluorofluorescein) (maximum intensity projections). (Scale bar, 100 µm.) (*E*) Immunofluorescence analysis of SHEAR devices (schematic on *Top Left*) after 3 d in culture, performed with arginase-1 (Arg-1, marker for hepatocytes), lectin (marker for HUVECs), and VE-Cad (marker for HUVEC tight junctions) (maximum intensity projections). (Scale bar, 500 µm.) The small bright green spots are from cell-death–related artifacts. (*F*) Albumin and urea concentrations are present in channels over 6 d in SHEAR devices with HUVECs, Hep spheroids, and fluid flow (*n* = 3 devices, mean ± SEM). (*G*) Cytochrome P450 (isoforms 3A4 and 2C9) induction by 72-h treatment with rifampicin (*n* = 3 devices, mean ± SEM, ****P < 0.0001, not significant [ns]: *P* > 0.05, two-way ANOVA with Sidak’s multiple comparisons test).

To support coculture of hepatocytes and endothelial cells in SHEAR devices, we screened a number of media conditions in static cultures and found a 50% vol/vol mixture of hepatocyte and endothelial media (H–E medium) to be optimal (*SI Appendix*, Fig. S2). To impart flow in a high throughout manner, devices were cultured on a rocker in a tissue culture incubator, which actuated devices to ±25° at a frequency of 1 Hz. This corresponds to a maximum shear stress of 6.21 dyn/cm^2^, with a mean magnitude of 3.95 dyn/cm^2^. This oscillatory, low-grade shear stress is similar to what is observed in sinusoids in vivo (38). Inlet and outlet ports were incorporated into the SHEAR devices and used to perfuse media through the HUVEC-seeded channel using a rocker platform. Upon application of flow, the vessel-like structure remained lumenized (Fig. 2*A*), demonstrated stable cell–cell junctions as evidenced by vascular endothelial cadherin (VE-cadherin) staining throughout the channel (Fig. 2*B* and Movie S1), and expressed primary cilia, based on the presence of acetylated α-tubulin (Fig. 2*C*). Furthermore, other expected flow-dependent changes were observed: Actin filaments underwent alignment in the direction of flow; nitric oxide release was increased (Fig. 2*D*); and gene expression of KLF2, NOS3, and COX-2 was elevated (*SI Appendix*, Fig. S3).

Under the application of flow, the devices displayed stable expression of both hepatic- and endothelial-specific markers in H–E medium. Albumin production, a proxy for Hep protein synthesis, increased over the course of a week (Fig. 2*F*), and urea production, a proxy for Hep nitrogen metabolism, remained stable during that period (Fig. 2*F*). As an indicator of xenobiotic-enhanced drug metabolism, treatment with rifampicin led to 10.3 (CYP3A4)- and 2.1 (CYP2C9)-fold inductions in cytochrome P450 enzymes (Fig. 2*G*). Furthermore, hepatic spheroids in the devices stably expressed the hepatocyte-specific marker Arg-1, and endothelial cells stably expressed VE-cadherin and stained positive for lectin (Fig. 2*E*).

### Acute Flow-Rate Changes Promote Secretion of Regeneration-Associated Factors.

**Fig. 3.**
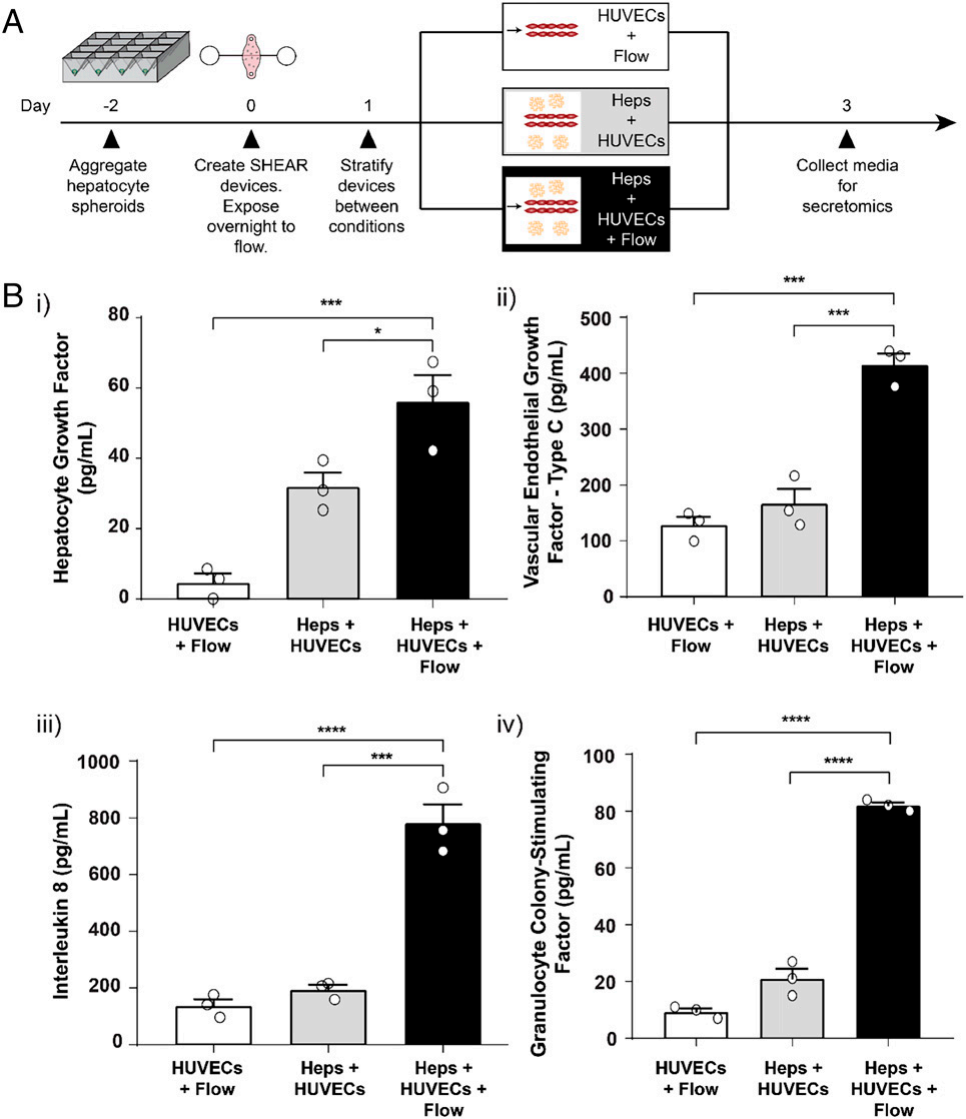
Application of flow promotes secretion of regeneration-associated factors. (*A*) Timeline for SHEAR device perturbation with flow and measurement of secreted factors. Media from devices were collected and assayed for secreted proteins using a multiplexed immunoassay. (*B*) Quantification of (*i*) HGF, (*ii*) VEGF-C, (*iii*) IL-8, and (*iv*) G-CSF production in flow-through media from the devices. (*n* = 3 devices, mean ± SEM, *P < 0.05, ***P < 0.001, ****P < 0.0001, one-way ANOVA with Tukey’s multiple comparisons test).

Having established that the SHEAR platform maintains survival and steady-state function of both endothelial and hepatic compartments, we sought to mimic a regenerative environment in order to assay for the production of secreted, soluble signals that have been reported to participate in human liver regeneration. Angiocrine signals derived from endothelial cells are key mediators of intercellular communication and play an important role in organ growth and regeneration (10, 17). In mice, it has been shown that post-PHx, there is an acute elevation in blood flow that corresponds with increased hepatocyte DNA synthesis (17, 39). Specifically, the elevated blood flow is associated with increased activation of mechanosensory molecules β1 integrin and vascular endothelial growth factor receptor 3 (VEGFR3) in liver endothelial cells, which leads to the secretion of HGF, a key regulator of liver growth (17). Thus, stretch of endothelial cells can act as a trigger for angiocrine signal production and increased proliferation and survival of hepatocytes. Based on these observations, we posited that a relevant model of liver regeneration must incorporate tunable fluid flow as an input. Consistent with this hypothesis, secretome analysis of the media flowing through the device revealed the presence of 13 endothelial- and hepatocyte-derived factors when the SHEAR device included the parenchymal compartment and was subjected to flow (*SI Appendix*, Fig. S4*B* and Fig. 3*B*). Nonetheless, minimal apparent DNA synthesis was observed within the cultured hepatocytes (*SI Appendix*, Fig. S4 *C* and *D*).

### Regenerative Cues Promote Primary Human Hepatocyte Cell-Cycle Entry in the SHEAR Device.

**Fig. 4.**
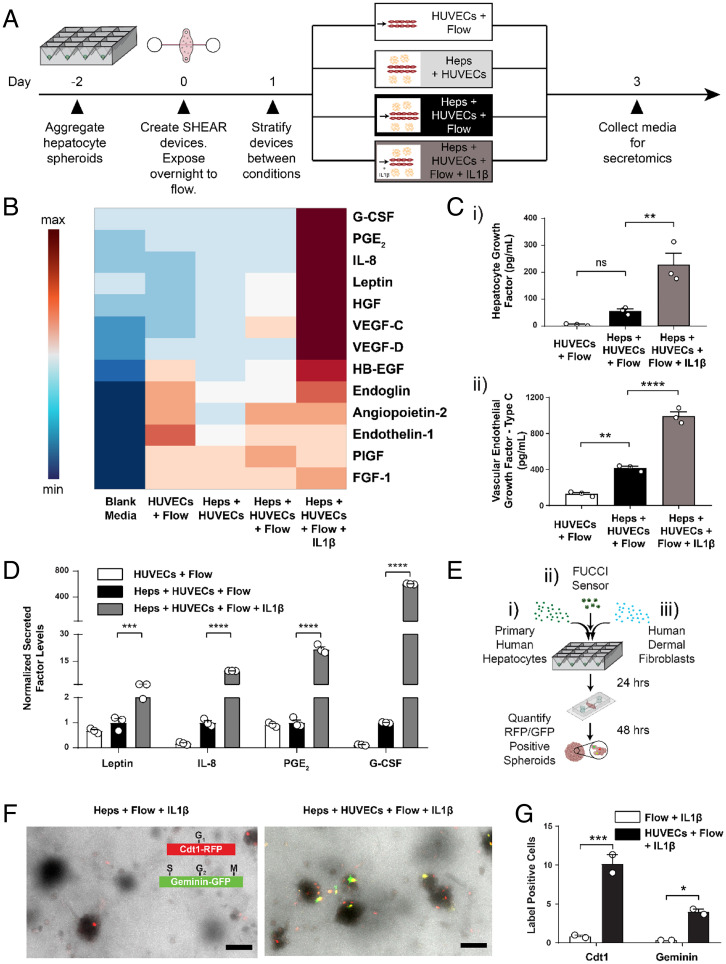
Mimicking a regenerative signal stimulates cell-cycle entry in primary human hepatocytes. (*A*) Timeline for SHEAR device perturbation with cytokines and assaying. Media from devices were collected and assayed for secreted proteins using a multiplexed immunoassay. (*B*) Row-normalized heatmap of candidate factors present in the flow-through media at day 3 (d3) under various device conditions. (*C*) Quantification of (*i*) HGF and (*ii*) VEGF-C production in flow-through media from the devices. (*D*) Quantification of the four candidate factors downselected using self-organizing maps. The factor levels are normalized to the “Heps + HUVECs + flow” condition. (*n* = 3 devices, mean ± SEM, **P < 0.01, ***P < 0.001, ****P < 0.0001, not significant [ns]: *P* > 0.05, one-way ANOVA with Tukey’s multiple comparisons test). (*E*) Timeline for assaying human hepatocyte proliferation inside the devices in 3D. The FUCCI sensor is transduced in the hepatocytes overnight, and then fibroblasts are added. (*F*) Immunofluorescence analysis of proliferation inside the devices, depicted via positive Cdt1 (a marker of G_1_ phase of the cell cycle) and geminin (a marker of S, G_2_, and M phases of the cell cycle) expression inside spheroids (maximum intensity projections). (Scale bar, 100 µm.) (*G*) Bar graph of the total cells in a field of view (FOV) that are positive for Cdt1 and/or geminin (*n* = 2 devices where each value is an average of three FOV, mean ± SEM, *P < 0.05, ***P < 0.001, two-way ANOVA with Tukey’s multiple comparisons test).

A modular, multiple-input platform like the SHEAR device provides us fine control over a complex in vivo phenomenon and gives us an opportunity to test the impact of different candidate soluble factors on regeneration. Beyond just correlations, we can determine the necessity and sufficiency of certain factors. We picked interleukin-1 beta (IL1β) as a candidate stimulatory factor to test, as it was found to be up-regulated in a 42-y-old human 1.5 h post-PHx (40) (*SI Appendix*, Fig. S5). First, we investigated the direct effect of IL1β on hepatocytes in the absence of endothelial cells. While we saw an increase in production of a few factors in the device’s circulating media, other relevant factors, including HGF and VEGF type C (VEGF-C), did not increase (*SI Appendix*, Table S1). Next, we tested whether IL1β can synergize with HUVECs to cause a regenerative response in hepatocytes. Upon application of IL1β to SHEAR devices populated with both Heps and HUVECs in the presence of flow, we detected an amplification of secreted factors, including but not limited to HGF, VEGF-C, PGE_2_, leptin, IL-8, and granulocyte colony stimulation factor (G-CSF) (Fig. 4 *B*–*D*). Notably, these responses appeared to be amplified by flow (*SI Appendix*, Table S2). By seeding the SHEAR devices with hepatocytes that had been modified with a FUCCI probe to read out their entry into the cell cycle (*SI Appendix*, Fig. S4*D*), we found that IL1β stimulation in the presence of HUVECs and flow led to increased cell-cycle entry of hepatocytes (Fig. 4 *F* and *G*). In contrast, IL1β exposure in the absence of HUVECs led to no detectable markers of cell cycling in hepatocytes. Therefore, we concluded that the IL1β-induced response was primarily mediated through HUVECs.

### Prostaglandin E_2_ Induces Cell-Cycle Entry in Primary Human Hepatocytes.

**Fig. 5.**
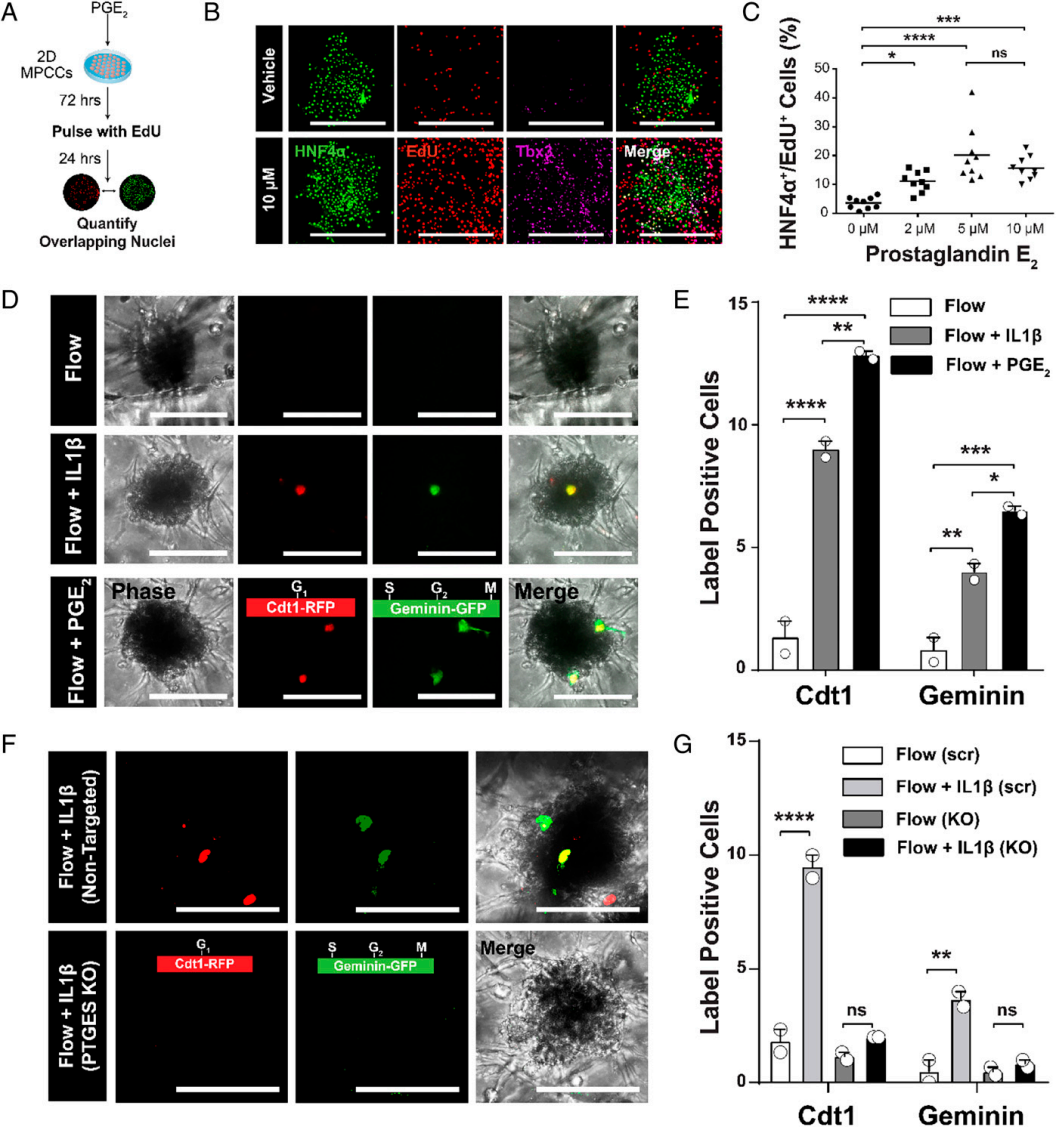
PGE_2_ promotes cell-cycle entry in primary human hepatocytes. (*A*) Timeline for assaying human hepatocyte cell-cycle entry in 2D. Varying concentrations (0, 2, 5, and 10 µM) of PGE_2_ were used. (*B*) Immunofluorescence analysis showing EdU incorporation and expression of T-box transcription factor (Tbx3) in Heps cultured in 2D when dosed with 10 µM PGE_2_ or with vehicle controls (maximum intensity projections). (Scale bar, 500 µm.) (*C*) Dot plot of the percentage of total cells in a well that are double positive for EdU and HNF4α. Each dot indicates the average of three islands inside a given well (*n* = 9 wells, *P < 0.05, ***P < 0.001, ****P < 0.0001, not significant [ns]: *P* > 0.05, two-way ANOVA with Tukey’s multiple comparisons test). (*D*) Immunofluorescence analysis of proliferation inside the devices, depicted via positive Cdt1 (a marker of G_1_ phase of the cell cycle) and geminin (a marker of S, G_2_, and M phases of the cell cycle) expression inside spheroids (maximum intensity projections). (Scale bar, 100 µm.) (*E*) Bar graph of the total cells in a FOV that are positive for Cdt1 and/or geminin (*n* = 2 devices where each value is an average of three FOV, mean ± SEM, *P < 0.05, **P < 0.01, ***P < 0.001, ****P < 0.0001, two-way ANOVA with Tukey’s multiple comparisons test). (*F*) Immunofluorescence analysis of proliferation inside devices with PTGES-knockout HUVECs and nontargeted controls, depicted via positive Cdt1 and Geminin expression inside spheroids (maximum intensity projections). (Scale bar, 100 µm.) (*G*) Bar graph of the total cells in a FOV that are positive for Cdt1 and/or geminin (*n* = 2 devices where each value is an average of three FOV, mean ± SEM, **P < 0.01, ****P < 0.0001, ns: *P* > 0.05, two-way ANOVA with Tukey’s multiple comparisons test).

To determine whether PGE_2_ stimulation is sufficient to induce cell-cycle entry in human hepatocytes, we tested it in a configuration without other confounding factors such as endothelial cells and flow. Previously, our laboratory had developed the micropatterned coculture (MPCC) platform, which consists of primary human hepatocytes organized into two-dimensional (2D) islands that are surrounded by supportive fibroblast cells (29). While this system has already been used to study hepatitis B (41), hepatitis C (42), malaria (43, 44), and liver metabolism (45, 46), here we utilized it as a testbed for studying human hepatocyte cell cycling (Fig. 5*A*). After 72 h of stimulation with varying concentrations of PGE_2_, we observed increased 5-ethynyl-2′-deoxyuridine (EdU) incorporation across human hepatocytes (Fig. 5*B* and *SI Appendix*, Fig. S6). Upon 10 µM PGE_2_ stimulation, the percentage of hepatocytes entering into the cell cycle increased from 5 to 20% in MPCCs, as indicated by EdU incorporation (Fig. 5*C*). While PGE_2_ can lead to cell-cycle entry in Heps when applied directly to cocultured cells in a simplified, 2D platform, we sought to characterize whether flow channel-borne PGE_2_ could also promote cell-cycle entry of PHH aggregates in 3D devices. Using the FUCCI circuit-expressing hepatocytes, we confirmed that PGE_2_ can elicit a similar effect on the primary human hepatocytes in our SHEAR devices (Fig. 5 *D* and E). As compared to control conditions, the number of label-positive hepatocytes significantly increased with both PGE_2_ and IL1β. A total of 10 µM PGE_2_, however, consistently induced a more robust cell-cycle entry readout than 10 ng/mL IL1β.

### Prostaglandin E_2_ Serves as Mediator of IL1β-Induced Cell-Cycle Entry in Primary Human Hepatocytes within the Device.

**Fig. 6.**
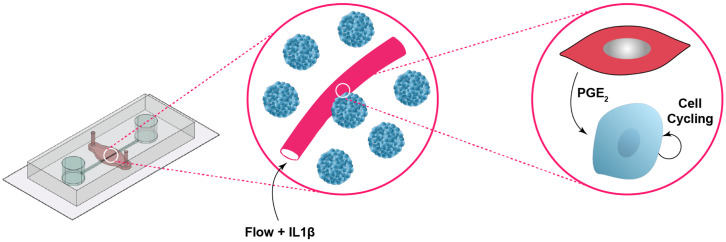
Model of molecular interactions in SHEAR. Cytokine stimulation combined with flow and IL1β stimulates endothelial cells to produce PGE_2_, which plays an important role in the signaling cascade that leads to primary human hepatocyte cell-cycle entry in SHEAR devices. Schematic figure is not drawn to scale.

Since paracrine interactions between hepatocytes and endothelial cells are extensive during liver regeneration (8, 10, 15), we hypothesized that IL1β stimulation leads to increased production of PGE_2_ by endothelial cells, which in turn is responsible for increased cell-cycle entry of hepatocytes. We first confirmed that IL1β stimulation does not drastically affect endothelial morphology and can stimulate HUVECs to produce PGE_2_ in the absence of Heps (*SI Appendix*, Fig. S7). Next, we exposed HUVECs to CRISPR-Cas9 lentiviral particles and guide RNA targeted against prostaglandin E synthase (PTGES), an enzyme necessary for the biosynthesis of PGE_2_ (47). After confirmation that PTGES expression was lost and PGE_2_ production was impaired in these endothelial cells (*SI Appendix*, Fig. S8), we incorporated them into hepatocyte-containing SHEAR devices and added IL1β to the endothelial channel. In the absence of PGE_2_ production, the IL1β-induced hepatocyte cell-cycle entry response was completely abrogated (Fig. 5 *F* and *G*). These data confirm that PGE_2_ is not only necessary for human hepatocyte cell cycling in a simplified 2D context, but it is also essential to initiate the Hep proliferative response in a 3D setting in the presence of IL1β and flow stimulation (Fig. 6).

## Discussion

In this study, we developed an organotypic in vitro model of human liver regeneration to investigate the impact of a small number of defined perturbations within a multicellular, functionally driven context. To accomplish this, we functionally mimicked key regeneration events that occur in the acute stages after a partial liver resection. By supplementing 3D hepatocyte platforms with an appropriate vascular niche and a necessary flow schema, our SHEAR model can be used to provide an improved understanding of the interplay between various components of human liver regeneration. While a recent model of a tubular liver structure also included a HUVEC-lined channel and investigated flow as an input, our device more directly examines the cross-talk between endothelial and parenchymal cells, and importantly, incorporates primary human hepatocytes rather than an immortalized hepatic cell line (48). In so doing, we were able to model flow-dependent angiocrine changes observed in vivo and also elucidate mechanistic signaling interactions that occur early in the regenerative process. Identifying factors that can promote PHH proliferation will be a critical component of improving current liver cell therapies, given the existing limited sources of proliferative human hepatocytes. While the liver has a remarkable capability to regenerate in vivo, this proliferative potential is often lost when hepatocytes are cultured in vitro (29, 49).

Although there are numerous changes that happen during liver regeneration in vivo, we focused on flow-, paracrine-, and cytokine-induced events in our platform. In this work, we integrated our 3D liver platform (35) with the vascular model developed by Polacheck et al. (50, 51). The integrated device has both liver parenchymal and endothelial vascular compartments, and through embedding both in fibrin, our biomaterial of choice, it allows for cross-talk between these compartments. Fibrin has innate angiogenic properties (37), which makes it a justifiable choice for this study; however, the human liver ECM consists of a mixture of fibronectin and various collagens (52). Even though there is diffusion and advection of soluble factors through fibrin, other natural and synthetic biomaterials may better represent physiological conditions and should be considered in future versions of the platform. While hepatic spheroids in the SHEAR platform preserve apical polarity, there exist key anatomic differences between the device and the hepatic microenvironment. For example, the distance between the basal aspect of the spheroids and the endothelial-lined vessels is not representative of the native liver.

Flow is an important aspect of regeneration biology. Prior work has suggested that early hemodynamic changes that arise following PHx are important and that these changes induce a global spectrum of events across the entire liver that resembles a wound healing response (53). Since the endothelial-lined channel that passes through the SHEAR device has a patent lumen, flow can be modulated through this compartment to study effects of shear stress and circumferential endothelial stretch on angiocrine factor production. These effects can be explored in both the presence and absence of circulating cytokines, allowing us to either combine the effects of flow with circulating factors or to subtract them. We modeled flow through the vascular channel via a rocker platform, which applies an oscillatory, low-grade shear force, similar to what is observed in sinusoids in vivo (38). We anticipate future evolutions of the platform to include a thorough evaluation of syringe pump-based flow, including its effects on PGE_2_ production. While our platform utilizes HUVECs as the model endothelial cells, the human liver harbors LSECs that line the sinusoidal capillary channels of the liver. The liver-specific endothelium establishes a specialized vascular niche that deploys growth factors (10). We anticipate future evolutions of the platform to incorporate LSECs and/or other organ-specific endothelial cells.

Lorenz et al. reported that in vivo blood perfusion promotes liver growth through mechanotransduction in liver endothelial cells and subsequent production of HGF (17). In our studies, we validated that SHEAR devices are capable of recapitulating this increased HGF production in vitro following the application of flow. In addition to HGF, we also depicted this phenomenon with a host of other angiocrine factors such as VEGF, Ang2, and others. A better understanding of how flow influences the various pathways involved in liver regeneration, and how those pathways intersect, may aid in uncovering molecular mechanisms at play in diseases such as cirrhosis, where fluid flow has been impaired. Furthermore, by uncovering candidate regenerative factors via SHEAR, we can put forward clinical strategies to improve cell therapies such as hepatocyte transplants and engineered liver grafts, and advance their use as alternatives to orthotopic liver transplantation (OLT). While an OLT is currently the primary treatment for end-stage liver disease and certain cancers, many challenges remain with this procedure, including donor organ shortages, a growing need for retransplantation, and adverse effects associated with long-term immunosuppression (54).

Nonetheless, flow does not act independently of other factors. Circulating soluble factors such as cytokines and growth factors are up-regulated and have been shown to be central to the regenerative response (5). To illustrate this concerted response, we introduced IL1β into our system. IL1β stimulated PHHs in SHEAR devices to undergo cell-cycle entry, and this cellular response was amplified by flow. Furthermore, endothelial cells were necessary to observe Hep cell-cycle entry in our system. Although cell-cycle entry is a necessary intermediary, practical assay limitations prevented us from assessing physical cell division in this study. Optically assessing percent increases in cell number in a spheroid format is challenging due to the light scattering nature of its 3D architecture. Future evolutions of the platform may permit more detailed and longer-term imaging assays that will enable visualization of explicit hepatocyte proliferation. A potential limitation of the SHEAR platform is its ability to identify nonhepatic or endothelial-derived regenerative factors such as epidermal growth factor (EGF), which is canonically produced in the Brunner’s glands. It is also possible that additional signals and paracrine interactions may be necessary to fully promote hepatocyte population expansion, and the microfluidic platform can certainly be expanded to accommodate studies designed to test these hypotheses. We also attempted to elucidate the factor(s) responsible for the observed flow-dependent cell-cycle initiation. Determination of individual factors that can supplement hepatocyte proliferation ex vivo is critical for augmenting cell numbers prior to therapeutic implantation. We identified PGE_2_ as a regenerative factor both in 2D and 3D human tissue cultures and as a mediator of the IL1β-induced response. Previous work implicating PGE_2_ in this pathway has not investigated its effect on primary human hepatocytes (55–57). In the future, treatment with PGE_2_ might be considered for use as a preconditioning agent for liver transplants, or PGE_2_-releasing depots could be incorporated into engineered grafts in order to promote the expansion of engrafted hepatocytes in vivo, even in the absence of an injury stimulus.

Through our studies, we show an application of the SHEAR device whereby it uncovers various axes of regeneration biology. Given its modularity, the model can be repurposed to study unexplored interactions and discover novel mediators that can help human hepatocytes expand ex vivo. For example, in the context of engineered liver grafts, very little is known about the vasculogenic and regenerative potential of various biomaterials. In the future, we anticipate that various synthetic and natural biomaterials will be investigated on our platform for their ability to induce graft regeneration, in addition to possibly broadening its uses to studying the effects of different vascular configurations and organ-specific niches. The platform can also be altered to study an acute injury response, which can be induced by hepatotoxins such as acetaminophen, genetic kill switches such as inducible caspase-9 (iCASP9) (58), or physical perturbations such as heat.

## Materials and Methods

### Fibroblast Culture.

Neonatal human dermal fibroblasts (Lonza) were purchased commercially. Murine embryonic 3T3-J2 fibroblasts were a gift from Howard Green, Harvard Medical School, Boston, MA. Both cell types were cultured at 37 °C, 5% CO_2_ in Dulbecco’s modified Eagle’s medium (DMEM, Corning Life Sciences) with high glucose, 10% (vol/vol) fetal bovine serum (Gemini Bio-Products), and 1% (vol/vol) penicillin/streptomycin (Corning Life Sciences). During maintenance, fibroblasts were passaged at 80% confluency and kept below passage 7 for all experiments.

### Endothelial Cell Culture.

Pooled HUVECs (Lonza) were purchased commercially. They were cultured at 37 °C, 5% CO_2_ in endothelial cell growth medium-2 (EGM-2) (Lonza). During maintenance, HUVECs were passaged at 80% confluency and kept below passage 5 for all experiments.

### Three-Dimensional Primary Human Hepatocyte-Fibroblast Aggregation.

Fibroblasts were growth arrested with mitomycin C (EMD Millipore) dissolved in fibroblast culture media at 10 μg/mL for 3 to 4 h at 37 °C. Six-well polystyrene plates containing pyramidal inserts were passivated using 5% pluronic (Sigma-Aldrich) for 30 min. Afterward, each well was rinsed three times with 2 mL DMEM containing 1% (vol/vol) penicillin/streptomycin per well. Media supplemented with insulin, transferrin, and selenous acid (ITS) was prepared from DMEM with L-glutamine supplemented with 1% (vol/vol) ITS Universal Culture Supplement (Corning Life Sciences), 1% (vol/vol) penicillin/streptomycin, 10% (vol/vol) fetal bovine serum, 15.4 mM Hepes (Thermo Fisher Scientific), 70 ng/mL glucagon (Sigma-Aldrich), and 40 ng/mL dexamethasone (Sigma-Aldrich). Cryopreserved Heps were thawed, spun down at 60 × *g* for 6 min in DMEM and resuspended in ITS media. Growth-arrested fibroblasts were washed several times with DMEM containing 1% (vol/vol) penicillin/streptomycin and then dissociated using 0.25% trypsin (Thermo Fisher). Fibroblasts were spun down at 1,000 rpm for 5 min and resuspended in ITS media. The cells were added to each well of the polystyrene plate containing pyramidal inserts in the following proportions: 0.6 M PHHs and 0.6 M fibroblasts in 2 mL of ITS media. The plate was then spun at 60 × *g* for 6 min and incubated at 37 °C, 5% CO_2_ for 2 d to allow the cells to aggregate.

### SHEAR Device Fabrication.

One-channel microfluidic devices were fabricated and assembled using photo and soft lithography as previously described (50, 51). After being plasma treated for 30 s at 100 W, the assembled devices were surface functionalized with 0.01% poly-L-lysine (Sigma-Aldrich) and 1% glutaraldehyde (Sigma-Aldrich) at room temperature for 5 min each to promote the binding of ECM to the device surface. The devices were then washed in water overnight at room temperature. On the day of cell seeding, each device was washed in 70% ethanol and inserted with a steel acupuncture needle (300 µm outer diameter, Hwato) followed by a 15-min ultraviolet (UV) sterilization. A solution of 2.5 mg/mL bovine fibrinogen (Sigma-Aldrich), 1 U/mL bovine thrombin (Sigma-Aldrich), and Dulbecco's phosphate-buffered saline (PBS) was added into the ECM chambers of the devices and allowed to cross-link at room temperature for 10 min before media addition. For conditions with hepatic spheroids, aggregates were added to the fibrinogen solution at 1.5 wells of aggregates/mL, and the devices were rotated during cross-linking to ensure uniform spheroid distribution. Needles were removed from the devices to form hollow microfluidic channels surrounded in fibrin. A suspension of HUVECs (Lonza) was added at 0.5 million cells/mL to the reservoirs connecting the microfluidic channels, and the cells were allowed to adhere to the top and bottom surfaces of the channels for 5 min each at 37 °C. Devices were then rinsed with fresh media to remove nonadherent cells and maintained at 37 °C on either a tilting rocker (5 rpm) for flow conditions or a flat surface for static conditions. After optimization of media, all experiments were conducted in media containing 50% (vol/vol) ITS and 50% (vol/vol) EGM-2 (H–E medium).

### Hepatocyte Function Assays.

Albumin was measured in collected cell culture supernatant using a commercial human albumin-specific enzyme-linked immunosorbent assay (ELISA) quantitation kit (Bethyl Laboratories). Urea was measured in collected cell culture supernatant using a commercial diacetylmonoxime-based blood urea nitrogen diagnostic kit (StanBio Laboratory). Relative CYP3A4 and CYP2C9 activity was determined using commercial live-cell luciferin-based luminescence assay kits (Promega Corporation). For CYP induction studies, rifampin (Sigma-Aldrich) was diluted to a concentration of 25 µM in H–E medium and devices were stimulated for 72 h, with media being replenished every 24 h. All assays were performed per manufacturer instructions and absorbance/luminescence measurements were done using a Tecan plate reader.

### Device Immunofluorescence.

The devices were fixed with 4% paraformaldehyde (PFA, Electron Microscopy Sciences) in PBS for 15 min at 37 °C on the rocker. The devices were then washed three times with PBS and permeabilized with 0.25% Triton X-100 (Sigma-Aldrich) for 15 min. After another three washes with PBS, the cells were blocked with 3% bovine serum albumin (BSA) (Sigma-Aldrich) in PBS at 4 °C overnight. Primary antibodies were diluted in the blocking solution and incubated in the devices at 4 °C overnight with rocking. The devices were then washed in PBS at 4 °C overnight. Secondary antibodies and Hoechst (Thermo Fisher Scientific) were diluted in the blocking solution and incubated in the devices at 4 °C overnight with rocking, followed by a PBS wash at 4 °C overnight. The stained devices were stored in PBS at 4 °C until imaging. For immunofluorescence imaging, the devices were place on a Yokogawa CSU-21/Zeiss Axiovert 200M inverted spinning disk microscope with a 10× air objective or 25× water-immersion objective and an Evolve EMCCD camera (Photometrics). Fluorescence images were adjusted for brightness/contrast and merged using ImageJ (NIH). Nitric Oxide secretion was assayed using 4-amino-5-methylamino-2′,7′-difluorofluorescein diacetate (DAF-FM diacetate) (Thermo Fisher Scientific) per manufacturer instructions.

### Antibodies.

Primary antibodies were purchased from the following sources and utilized at the following dilutions: VE-cadherin (F-8, Santa Cruz Biotechnology; 1:200), Arginase-1 (Sigma-Aldrich; 1:400), acetylated α-tubulin (6-11B-1, Santa Cruz Biotechnology; 1:100), and HNF4α (C-19, Santa Cruz Biotechnology; 1:400). Dylight 649-conjugated Ulex Europaeus Agglutinin I lectin (1:200) was purchased from Vector Laboratories. For secondary antibodies, Alexa Fluor 488, 568, 594, and 647 anti-mouse, anti-goat, and anti-rabbit IgG secondary antibodies were purchased from Life Technologies.

### Gene Expression.

Cells in devices were lysed and homogenized in TRIzol (Thermo Fisher Scientific) after media removal. Total RNA was isolated via chloroform extraction and purified using the RNeasy MinElute Cleanup Kit (QIAGEN). cDNA synthesis was performed using the iScript cDNA synthesis kit (Bio-Rad) and quantitative PCR was carried out using the Taqman gene expression assay system (Thermo Fisher Scientific) in a BioRad CFX96 Real-Time System according to the manufacturer’s instructions. The FAM-labeled Taqman probes (Thermo Fisher Scientific) used were as follows: KLF2 (Hs00360439_g1), NOS3 (Hs01574659_m1), and COX-2 (Hs00153133_m1). Relative mRNA quantification was calculated with the ΔΔCt method, using a GAPDH probe as housekeeping gene.

### Secreted Factor Assays.

At the end of the experiment, supernatant was collected from device channels and stored at −80 °C until further analysis. For most secreted factors, the assays were done using a multiplexed Luminex bead-based assay by Eve Technologies. Frozen samples were shipped overnight on dry ice. However, for prostaglandin E_2_, the assay was conducted in-house using a prostaglandin E_2_ ELISA Kit (Cayman Chemical) according to manufacturer instructions.

### Cell-Cycle Quantification.

To quantify cycling hepatocytes, we utilized either 5-ethynyl-2′-deoxyuridine (EdU, Thermo Fisher Scientific) or a Premo FUCCI Cell Cycle Sensor (Thermo Fisher Scientific).

The FUCCI cell-cycle sensor enables a live imaging assay that consists of a fluorescent system that employs both a red (RFP) and a green (GFP) fluorescent protein fused to different regulators of the cell cycle: cdt1 and geminin. In the G1 phase of the cell cycle, geminin is degraded; therefore, only cdt1 tagged with RFP is present and appears as red fluorescence. In the S, G2, and M phases, cdt1 is degraded and only geminin tagged with GFP remains, resulting in cells with green fluorescence. During the G1/S transition, both proteins are present, and the cells appear with yellow fluorescence.

EdU is a nucleoside analog to thymidine and is incorporated into DNA during active DNA synthesis or cells in the S phase of cell-cycle progression. Once EdU is incorporated into the cells, it can be detected after fixation and permeabilization through a copper-catalyzed “click” reaction.

For 2D studies, Heps were cultured along with 3T3-J2 murine fibroblasts in MPCC configurations, as previously described (29, 30, 44). At the start of cell-cycle quantification, MPCC cultures were stimulated with 10 µM EdU for a period of 24 h. Then, each well was fixed with 4% PFA in PBS for 15 min, washed three times with PBS, permeabilized with 0.1% Triton X-100 for 10 min, washed three times with PBS and subsequently blocked for 1 h with 3% BSA in PBS. EdU staining was performed according to manufacturer instructions and wells were incubated overnight with HNF4α (1:400) in blocking buffer at 4 °C. After a 3× PBS wash, they were incubated for 1 h with Alexa Fluor 594 anti-goat IgG secondary antibody (1:1,000) and Hoechst in PBS at room temperature. After a final 3× PBS wash, the wells were imaged using a confocal microscope (Olympus FV1200) and overlapping nuclei (EdU and HNF4α) were quantified using ImageJ.

For 3D studies, the SHEAR devices were cultured as described in a previous section with a small modification to the protocol. Instead of aggregating both the PHHs and the fibroblasts at the same time, the Heps were first preaggregated and transduced with FUCCI at an estimated multiplicity of infection (MOI) of 5 in the microwells for 24 h in ITS media. Afterward, they were washed twice with DMEM media and aggregated along with mitomycin C–treated HDFs for another 24 h in ITS media. This was done to ensure that only Heps were transduced with the cell-cycle sensor. At the end of the experiment, the devices were fixed and imaged using a confocal microscope (Leica SP8) and geminin-GFP/Cdt1-RFP was quantified using ImageJ.

### Knockout Studies.

To create PTGES knockout (KO) HUVECs, lentiviral particles harboring CRISPR-Cas9, gRNAs targeting exon 1 of PTGES, and a puromycin resistance marker (purchased from Sigma-Aldrich) were utilized. HUVECs were plated in six-well tissue culture plates at 40,000 per well. The next day, lentiviral particles were added at an estimated MOI of 5 in HUVEC media containing 8 µg/mL hexadimethrine bromide (Polybrene, Sigma-Aldrich). After 48 h of transduction, wells were refreshed with normal HUVEC media. Puromycin (Sigma-Aldrich) was added to the wells 24 h after the media change at a concentration of 2 µg/mL in HUVEC media. The cells were passaged twice in puromycin-supplemented media and cryopreserved before further use.

### Experimental Replicates.

All averages and SDs shown are from at least three stand-alone experimental replicates. Furthermore, each experiment was repeated at least twice and trends were confirmed to be consistent across experiments.

## Supplementary Material

Supplementary File

Supplementary File

## Data Availability

The human PHx data are from a public dataset (Gene Expression Omnibus accession no. GSE15239) (40) and represent the transcriptome of a 42-y-old human who underwent PHx. The raw images that support the findings of this study are available from the corresponding author upon reasonable request. All other study data are included in the article and/or supporting information.

## References

[1] G. K. Michalopoulos, M. C. DeFrances, Liver regeneration. Science 276, 60–66 (1997). 9082986 10.1126/science.276.5309.60

[2] N. Fausto, J. S. Campbell, K. J. Riehle, Liver regeneration. Hepatology 43 (suppl. 1) S45–S53 (2006). 16447274 10.1002/hep.20969

[3] R. Taub, Liver regeneration: from myth to mechanism. Nat. Rev. Mol. Cell Biol. 5, 836–847 (2004). 15459664 10.1038/nrm1489

[4] G. M. Higgins, Experimental pathology of the liver; I. Restoration of the liver of the white rat following partial surgical removal. Arch. Pathol. (Chic.) (1931).

[5] N. Fausto, A. D. Laird, E. M. Webber, Liver regeneration. 2. Role of growth factors and cytokines in hepatic regeneration. FASEB J. 9, 1527–1536 (1995). 8529831 10.1096/fasebj.9.15.8529831

[6] J. M. Schoen, H. H. Wang, G. Y. Minuk, W. W. Lautt, Shear stress-induced nitric oxide release triggers the liver regeneration cascade. Nitric Oxide 5, 453–464 (2001). 11587560 10.1006/niox.2001.0373

[7] B. Z. Stanger, L. Greenbaum, The role of paracrine signals during liver regeneration. Hepatology 56, 1577–1579 (2012). 23038651 10.1002/hep.25911 PMC4671199

[8] B.-S. Ding , Inductive angiocrine signals from sinusoidal endothelium are required for liver regeneration. Nature 468, 310–315 (2010). 21068842 10.1038/nature09493 PMC3058628

[9] L. Boulter , Macrophage-derived Wnt opposes Notch signaling to specify hepatic progenitor cell fate in chronic liver disease. Nat. Med. 18, 572–579 (2012). 22388089 10.1038/nm.2667 PMC3364717

[10] S. Rafii, J. M. Butler, B.-S. Ding, Angiocrine functions of organ-specific endothelial cells. Nature 529, 316–325 (2016). 26791722 10.1038/nature17040 PMC4878406

[11] Q. Tan , The role of IL-1 family members and Kupffer cells in liver regeneration. BioMed Res. Int. 2016, 6495793 (2016). 27092311 10.1155/2016/6495793 PMC4820608

[12] B. D. Cosgrove , An inducible autocrine cascade regulates rat hepatocyte proliferation and apoptosis responses to tumor necrosis factor-alpha. Hepatology 48, 276–288 (2008). 18536058 10.1002/hep.22335 PMC4327877

[13] R. H. Waterston ; Mouse Genome Sequencing Consortium, Initial sequencing and comparative analysis of the mouse genome. Nature 420, 520–562 (2002). 12466850 10.1038/nature01262

[14] J. Mestas, C. C. W. Hughes, Of mice and not men: Differences between mouse and human immunology. J. Immunol. 172, 2731–2738 (2004). 14978070 10.4049/jimmunol.172.5.2731

[15] B.-S. Ding , Divergent angiocrine signals from vascular niche balance liver regeneration and fibrosis. Nature 505, 97–102 (2014). 24256728 10.1038/nature12681 PMC4142699

[16] W. Goessling , Genetic interaction of PGE2 and Wnt signaling regulates developmental specification of stem cells and regeneration. Cell 136, 1136–1147 (2009). 19303855 10.1016/j.cell.2009.01.015 PMC2692708

[17] L. Lorenz , Mechanosensing by β1 integrin induces angiocrine signals for liver growth and survival. Nature 562, 128–132 (2018). 30258227 10.1038/s41586-018-0522-3

[18] H. Hu , Long-term expansion of functional mouse and human hepatocytes as 3D organoids. Cell 175, 1591–1606 (2018). 30500538 10.1016/j.cell.2018.11.013

[19] C. Xiang , Long-term functional maintenance of primary human hepatocytes in vitro. Science 364, 399–402 (2019). 31023926 10.1126/science.aau7307

[20] L. Broutier , Culture and establishment of self-renewing human and mouse adult liver and pancreas 3D organoids and their genetic manipulation. Nat. Protoc. 11, 1724–1743 (2016). 27560176 10.1038/nprot.2016.097

[21] P. V. Moghe , Culture matrix configuration and composition in the maintenance of hepatocyte polarity and function. Biomaterials 17, 373–385 (1996). 8745335 10.1016/0142-9612(96)85576-1

[22] C. H. Cho, F. Berthiaume, A. W. Tilles, M. L. Yarmush, A new technique for primary hepatocyte expansion in vitro. Biotechnol. Bioeng. 101, 345–356 (2008). 18465801 10.1002/bit.21911 PMC4487520

[23] F. Goulet, C. Normand, O. Morin, Cellular interactions promote tissue-specific function, biomatrix deposition and junctional communication of primary cultured hepatocytes. Hepatology 8, 1010–1018 (1988). 2458307 10.1002/hep.1840080506

[24] O. Morin, C. Normand, Long-term maintenance of hepatocyte functional activity in co-culture: Requirements for sinusoidal endothelial cells and dexamethasone. J. Cell. Physiol. 129, 103–110 (1986). 3531216 10.1002/jcp.1041290115

[25] F. Berthiaume, P. V. Moghe, M. Toner, M. L. Yarmush, Effect of extracellular matrix topology on cell structure, function, and physiological responsiveness: Hepatocytes cultured in a sandwich configuration. FASEB J. 10, 1471–1484 (1996). 8940293 10.1096/fasebj.10.13.8940293

[26] J. C. Dunn, R. G. Tompkins, M. L. Yarmush, Long-term in vitro function of adult hepatocytes in a collagen sandwich configuration. Biotechnol. Prog. 7, 237–245 (1991). 1367596 10.1021/bp00009a007

[27] C. M. Ryan , Isolation and long-term culture of human hepatocytes. Surgery 113, 48–54 (1993). 8417488

[28] M. Huch , Long-term culture of genome-stable bipotent stem cells from adult human liver. Cell 160, 299–312 (2015). 25533785 10.1016/j.cell.2014.11.050 PMC4313365

[29] S. R. Khetani, S. N. Bhatia, Microscale culture of human liver cells for drug development. Nat. Biotechnol. 26, 120–126 (2008). 18026090 10.1038/nbt1361

[30] S. March , Micropatterned coculture of primary human hepatocytes and supportive cells for the study of hepatotropic pathogens. Nat. Protoc. 10, 2027–2053 (2015). 26584444 10.1038/nprot.2015.128 PMC5867906

[31] P. J. Lee, P. J. Hung, L. P. Lee, An artificial liver sinusoid with a microfluidic endothelial-like barrier for primary hepatocyte culture. Biotechnol. Bioeng. 97, 1340–1346 (2007). 17286266 10.1002/bit.21360

[32] T. J. Long , Modeling therapeutic antibody-small molecule drug-drug interactions using a three-dimensional perfusable human liver coculture platform. Drug Metab. Dispos. 44, 1940–1948 (2016). 27621203 10.1124/dmd.116.071456 PMC5118635

[33] J. Shan , Identification of small molecules for human hepatocyte expansion and iPS differentiation. Nat. Chem. Biol. 9, 514–520 (2013). 23728495 10.1038/nchembio.1270 PMC3720805

[34] Y. Du , Mimicking liver sinusoidal structures and functions using a 3D-configured microfluidic chip. Lab Chip 17, 782–794 (2017). 28112323 10.1039/c6lc01374k

[35] K. R. Stevens , InVERT molding for scalable control of tissue microarchitecture. Nat. Commun. 4, 1847 (2013). 23673632 10.1038/ncomms2853 PMC3660041

[36] K. R. Stevens , In situ expansion of engineered human liver tissue in a mouse model of chronic liver disease. Sci. Transl. Med. 9, eaah5505 (2017). 28724577 10.1126/scitranslmed.aah5505 PMC5896001

[37] J. Ceccarelli, A. J. Putnam, Sculpting the blank slate: How fibrin’s support of vascularization can inspire biomaterial design. Acta Biomater. 10, 1515–1523 (2014). 23933102 10.1016/j.actbio.2013.07.043 PMC3864148

[38] P. J. MacPhee, E. E. Schmidt, A. C. Groom, Intermittence of blood flow in liver sinusoids, studied by high-resolution in vivo microscopy. Am. J. Physiol. 269, G692–G698 (1995). 7491960 10.1152/ajpgi.1995.269.5.G692

[39] T. Niiya , Immediate increase of portal pressure, reflecting sinusoidal shear stress, induced liver regeneration after partial hepatectomy. J. Hepatobiliary Pancreat. Surg. 6, 275–280 (1999). 10526063 10.1007/s005340050118

[40] T. Galit, G. Eithan, Expression data from human livers shortly following partial hepatectomy. Gene Expression Omnibus. https://www.ncbi.nlm.nih.gov/geo/query/acc.cgi?acc=GSE15239. Accessed 1 March 2018.

[41] A. Shlomai , Modeling host interactions with hepatitis B virus using primary and induced pluripotent stem cell-derived hepatocellular systems. Proc. Natl. Acad. Sci. U.S.A. 111, 12193–12198 (2014). 25092305 10.1073/pnas.1412631111 PMC4143014

[42] A. Ploss , Persistent hepatitis C virus infection in microscale primary human hepatocyte cultures. Proc. Natl. Acad. Sci. U.S.A. 107, 3141–3145 (2010). 20133632 10.1073/pnas.0915130107 PMC2840339

[43] N. Gural , In vitro culture, drug sensitivity, and transcriptome of plasmodium vivax hypnozoites. Cell Host Microbe 23, 395–406 (2018). 29478773 10.1016/j.chom.2018.01.002 PMC8048090

[44] S. March , A microscale human liver platform that supports the hepatic stages of *Plasmodium falciparum* and vivax. Cell Host Microbe 14, 104–115 (2013). 23870318 10.1016/j.chom.2013.06.005 PMC3780791

[45] C. Lin, J. Shi, A. Moore, S. R. Khetani, Prediction of drug clearance and drug-drug interactions in microscale cultures of human hepatocytes. Drug Metab. Dispos. 44, 127–136 (2016). 26452722 10.1124/dmd.115.066027

[46] S. R. Khetani , Use of micropatterned cocultures to detect compounds that cause drug-induced liver injury in humans. Toxicol. Sci. 132, 107–117 (2013). 23152190 10.1093/toxsci/kfs326

[47] C. Jegerschöld , Structural basis for induced formation of the inflammatory mediator prostaglandin E2. Proc. Natl. Acad. Sci. U.S.A. 105, 11110–11115 (2008). 18682561 10.1073/pnas.0802894105 PMC2516235

[48] N. Mori, Y. Akagi, Y. Imai, Y. Takayama, Y. S. Kida, Fabrication of perfusable vascular channels and capillaries in 3D liver-like tissue. Sci. Rep. 10, 5646 (2020). 32286353 10.1038/s41598-020-62286-3 PMC7156376

[49] S. Wang, D. Nagrath, P. C. Chen, F. Berthiaume, M. L. Yarmush, Three-dimensional primary hepatocyte culture in synthetic self-assembling peptide hydrogel. Tissue Eng. Part A 14, 227–236 (2008). 18333775 10.1089/tea.2007.0143

[50] W. J. Polacheck , A non-canonical Notch complex regulates adherens junctions and vascular barrier function. Nature 552, 258–262 (2017). 29160307 10.1038/nature24998 PMC5730479

[51] W. J. Polacheck, M. L. Kutys, J. B. Tefft, C. S. Chen, Microfabricated blood vessels for modeling the vascular transport barrier. Nat. Protoc. 14, 1425–1454 (2019). 30953042 10.1038/s41596-019-0144-8 PMC7046311

[52] A. Martinez-Hernandez, P. S. Amenta, The hepatic extracellular matrix. I. Components and distribution in normal liver. Virchows Arch. A Pathol. Anat. Histopathol. 423, 1–11 (1993). 8212529 10.1007/BF01606425

[53] G. K. Michalopoulos, Liver regeneration. J. Cell. Physiol. 213, 286–300 (2007). 17559071 10.1002/jcp.21172 PMC2701258

[54] A. Zarrinpar, R. W. Busuttil, Liver transplantation: Past, present and future. Nat. Rev. Gastroenterol. Hepatol. 10, 434–440 (2013). 23752825 10.1038/nrgastro.2013.88

[55] Y. S. Mohamed, R. M. Abdelsalam, A. S. Attia, M. T. Abdel-Aziz, D. M. El-Tanbouly, Regulation of liver regeneration by prostaglandin E_2_ and thromboxane A_2_ following partial hepatectomy in rats. Naunyn Schmiedebergs Arch. Pharmacol. 393, 1437–1446 (2020). 32162076 10.1007/s00210-020-01848-8

[56] J. Xiao , Cyclooxygenase-1 serves a vital hepato-protective function in chemically induced acute liver injury. Toxicol. Sci. 143, 430–440 (2015). 25432964 10.1093/toxsci/kfu244

[57] M. Chakkour, S. Kreydiyyeh, FTY720P upregulates the na+/k+ atpase in hepg2 cells by activating S1PR3 and inducing PGE2 release. Cell. Physiol. Biochem. 53, 518–531 (2019). 31502430 10.33594/000000155

[58] A. X. Chen , Controlled apoptosis of stromal cells to engineer human microlivers. Adv. Funct. Mater. 30, 1910442 (2020). 33776613 10.1002/adfm.201910442 PMC7996305

