# Supplementary material for: A vascularized model of the human liver mimics regenerative responses
Source: Proc Natl Acad Sci U S A. 2022 Jun 28;119(28):e2115867119. doi: 10.1073/pnas.2115867119 (PMC9282349; doi:10.1073/pnas.2115867119)
Supplement: Supplementary File [file pnas.2115867119.sapp.pdf]

Supplementary Information for
A vascularized model of the human liver mimics regenerative responses

Arnav Chhabra, H.-H. Greco Song, Katarzyna A. Grzelak, William J. Polacheck, Heather E. Fleming, Christopher S. Chen, Sangeeta N. Bhatia

Sangeeta N. Bhatia
Email: sbhatia@mit.edu

This PDF file includes:

Figures S1 to S11
Tables S1 to S2
Legend for Video S1

Other supplementary materials for this manuscript include the following:

Video S1

Supplemental Figures

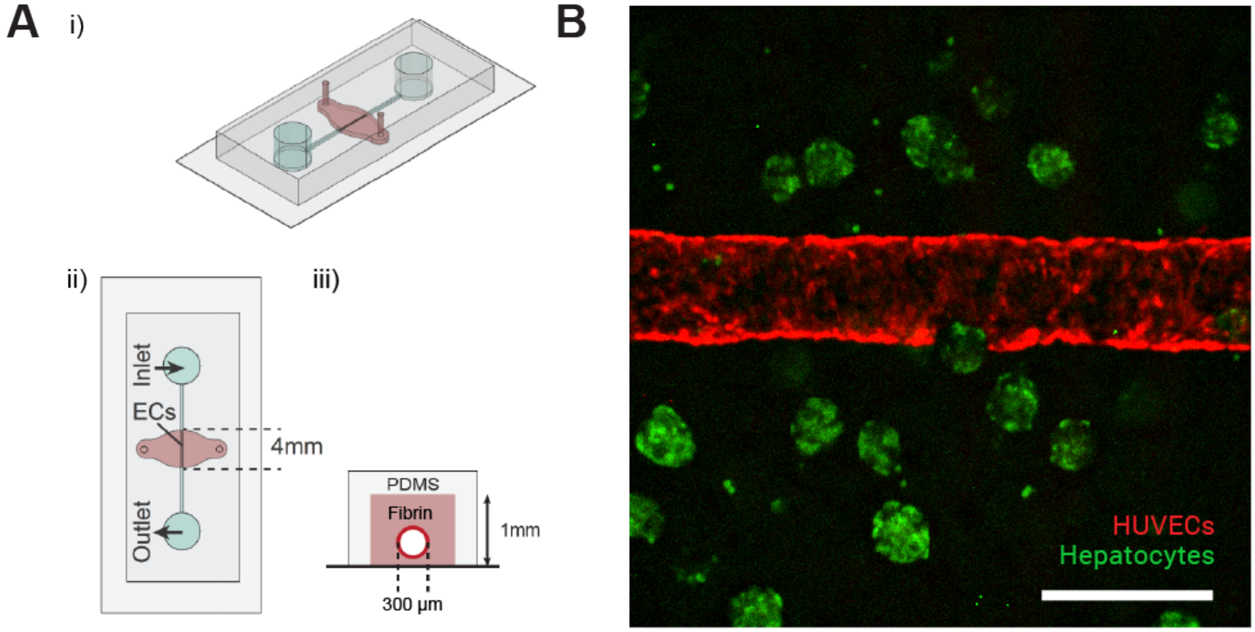

Figure S1

SHEAR devices combine a hepatic and vascular compartment. (**A**) A three-dimensional CAD rendering of the device, noting key dimensions and compartments. (**B**) Confocal imaging of a device after seven days in culture with flow, depicting hepatocyte spheroids in green and endothelial channel in red (maximum intensity projection, scale bar = 500 μm).

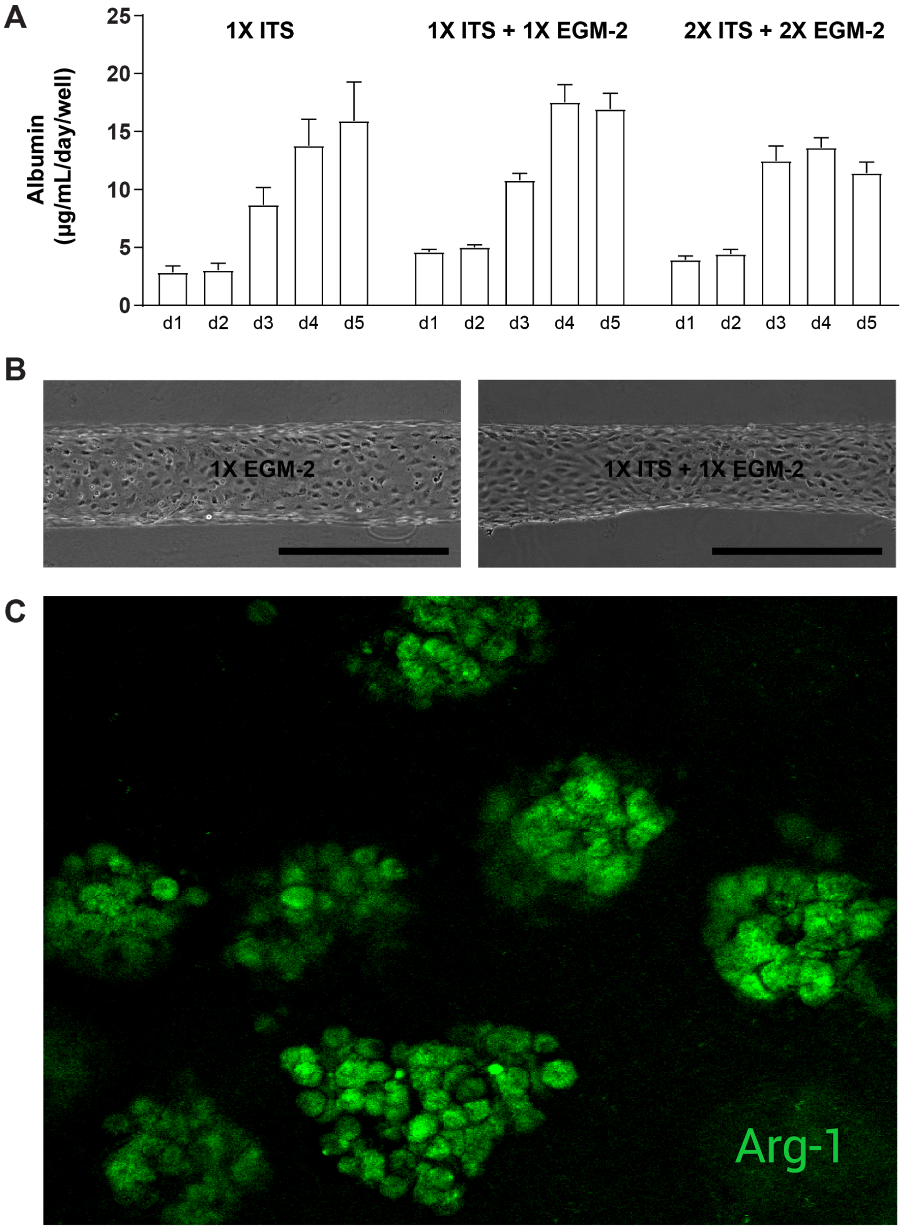

Figure S2

50% v/v mixture of hepatocyte (ITS) and endothelial (EGM-2) media (H-E media) supports hepatocyte and endothelial functionality. (**A**) Albumin concentrations as a function of time (days) from 3D fibrin gels harboring Hep spheroids (n = 3 gels, mean ± SEM). (**B**) Phase contrast images of endothelial channels, cultured for three days under different media conditions (scale bar = 500 µm). (**C**) Confocal imaging of a device after seven days in culture with flow, depicting Arginase-1 (Arg-1) expression in hepatic spheroids. (maximum intensity projection).

Video S1

Confocal z-stack scan of a device cultured under flow in H-E media for three days, depicting VE-Cadherin staining (red) and a cell nucleus marker (blue) (scale bar = 100 μm).

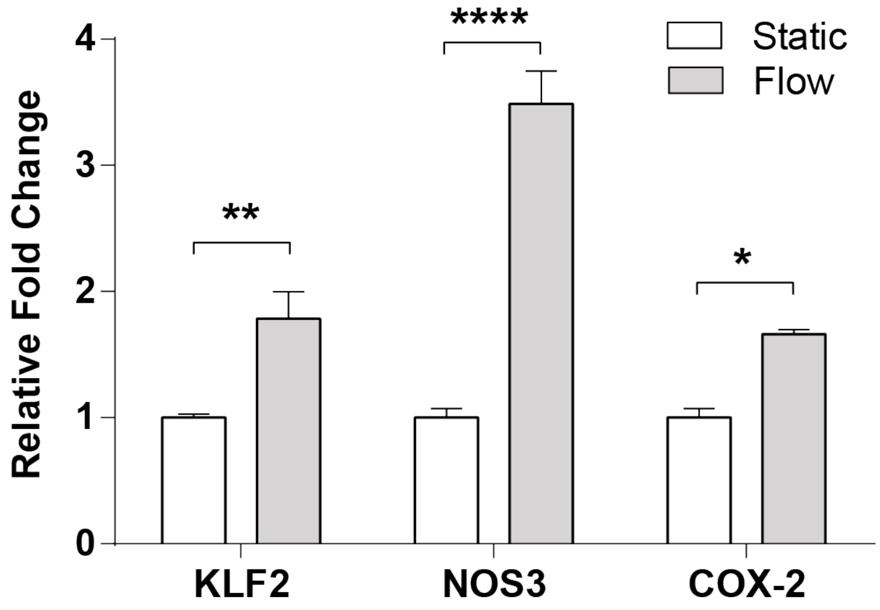

Figure S3

Bulk quantitative PCR analysis of various flow-dependent genes in the endothelial channel of devices cultured under flow or static conditions for three days (n = 3 devices, normalized to GAPDH, mean ± SEM).

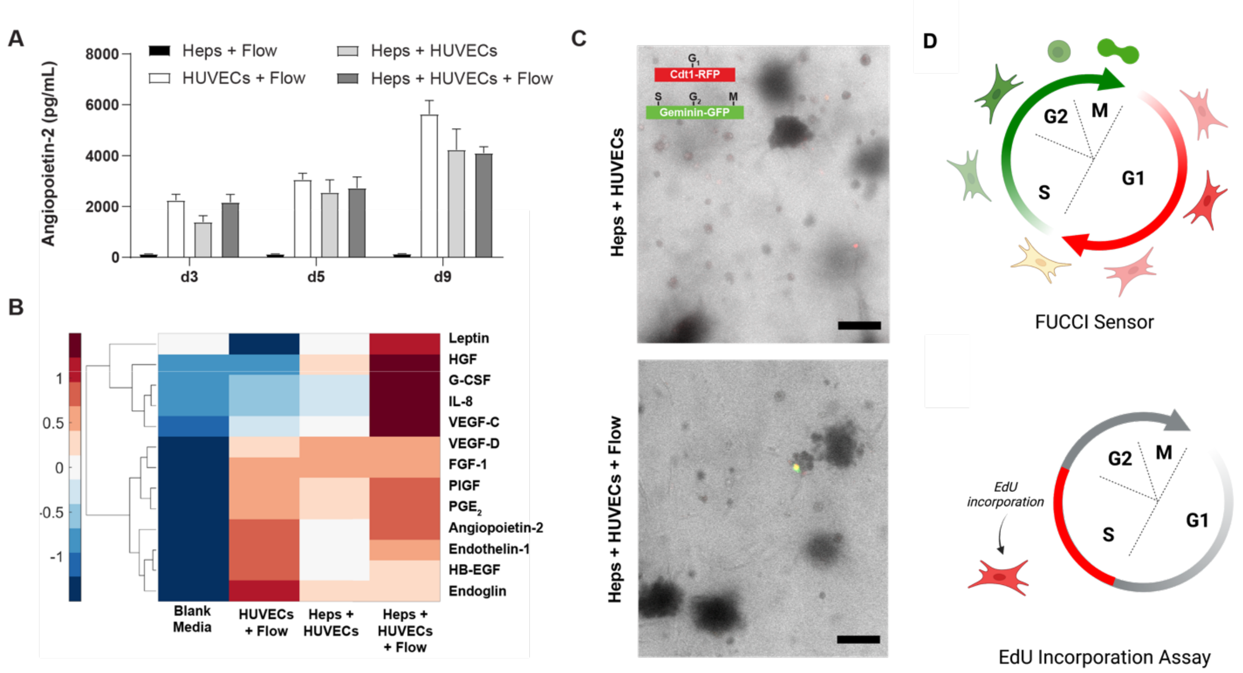

Figure S4

Application of flow promotes secretion of angiocrine factors, but does not stimulate appreciable cell cycle entry in Heps. (**A**) Quantification of Ang-2 in the flow-through media at various time points (n = 3 devices, mean ± SEM). (**B**) Row-normalized heatmap of candidate factors present in the flow-through media at d3 under various device conditions. (**C**) Immunofluorescence analysis of entry into cell cycle inside the devices, depicted via positive Cdt1 (marker of G_1 phase of cell cycle) and Geminin (marker of S, G_2 and M phases of cell cycle) expression inside spheroids (maximum intensity projections, scale bar = 100 µm). (**D**) Schematic describing the FUCCI cell cycle sensor and the EdU incorporation assay.

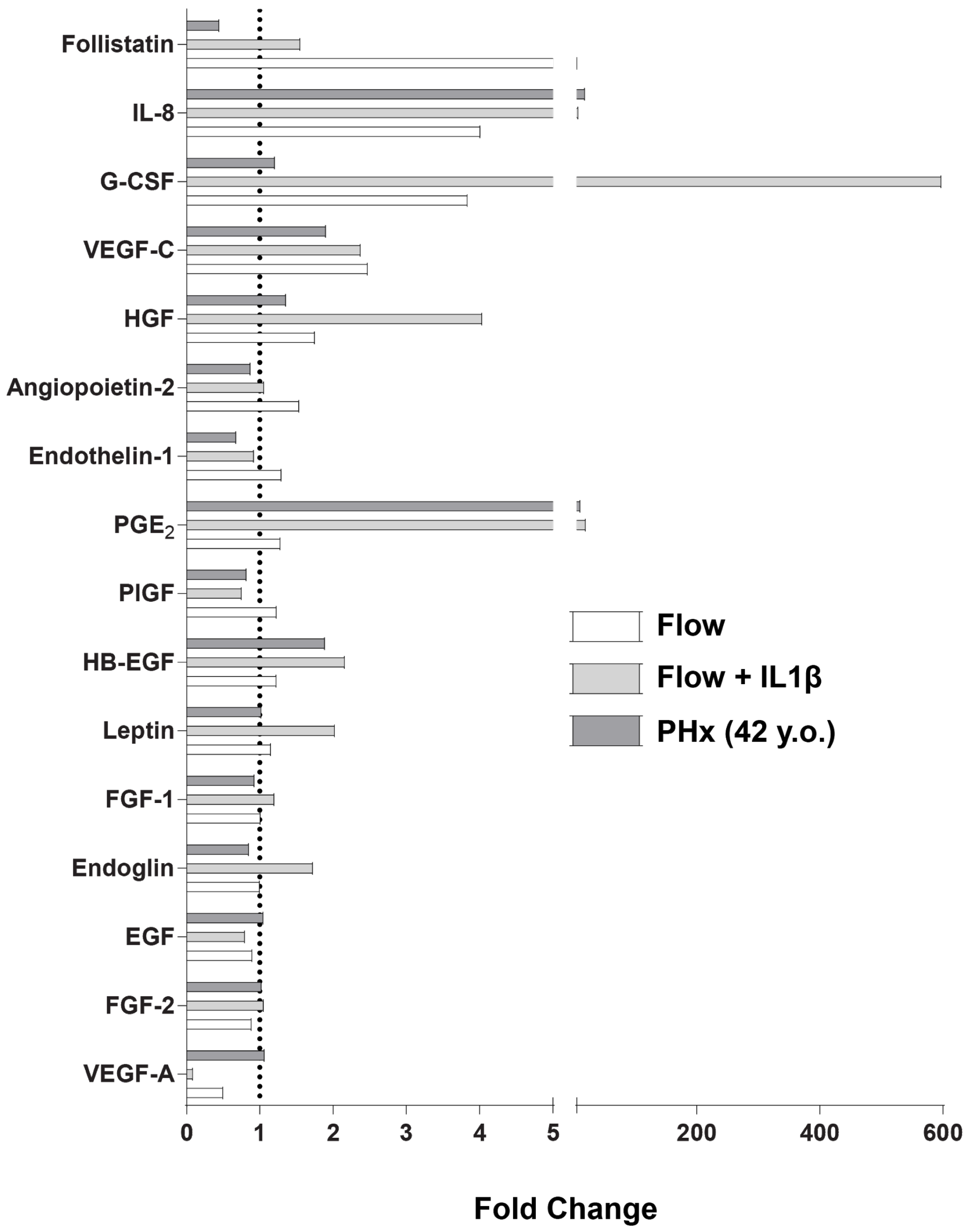

Figure S5

Flow and cytokine stimulation provides comparable induction of many factors to human PHx. Presented is a bar graph comparing relative induction of factors through various stimuli. Each is normalized to its control. The PHx data is from a public dataset (GEO accession # GSE15239) and represents the transcriptome a 42 yr old human who underwent PHx. Samples were collected before PHx and 1.5 hrs after PHx.

While not shown in the figure, IL1B is upregulated 3.78-fold and COX-2 (key mediator of PGE_2 biosynthesis) is upregulated 13-fold in the human, 1.5 hrs after PHx.

Table S1

Concentrations of various factors measured in the effluent of the devices cultured under various conditions for three days. All units are pg/mL.

	Heps	Heps + IL1β	(Heps + IL1β)/Heps
**Angiopoietin-2**	133	145.67	1.10
**Endoglin**	19	40.12	2.16
**Endothelin-1**	4	14.90	3.43
**FGF-1**	14	5.03	0.37
**G-CSF**	87	35152.49	403.73
**HB-EGF**	2	7.23	3.54
**HGF**	66	62.67	0.95
**IL-8**	980	968.33	0.99
**Leptin**	522	352.56	0.68
**PIGF**	104	923.89	8.85
**VEGF-C**	917	682.07	0.74
**VEGF-D**	4	11.51	2.83
**PGE_2**	50	50.33	1.01

Table S2

Concentrations of various factors measured in the effluent of the devices cultured under various conditions for three days. All units are pg/mL. The fourth column represents the ratio of the two columns.

	Heps + HUVECs + IL1β [1]	Heps + HUVECs + Flow + IL1β [2]	Ratio [2]/[1]
**Angiopoietin-2**	1539.25	4811.91	3.13
**Endoglin**	96.81	128.49	1.33
**Endothelin-1**	202.64	302.33	1.49
**FGF-1**	3.89	3.72	0.95
**G-CSF**	25967.68	34181.95	1.32
**HB-EGF**	9.95	20.57	2.07
**HGF**	93.26	1090.31	11.69
**IL-8**	5249.91	5366.14	1.02
**Leptin**	299.87	381.89	1.27
**PIGF**	87.40	142.70	1.63
**VEGF-C**	235.05	752.30	3.2
**VEGF-D**	8.50	7.62	0.9
**PGE_2**	470.00	1104.33	2.35

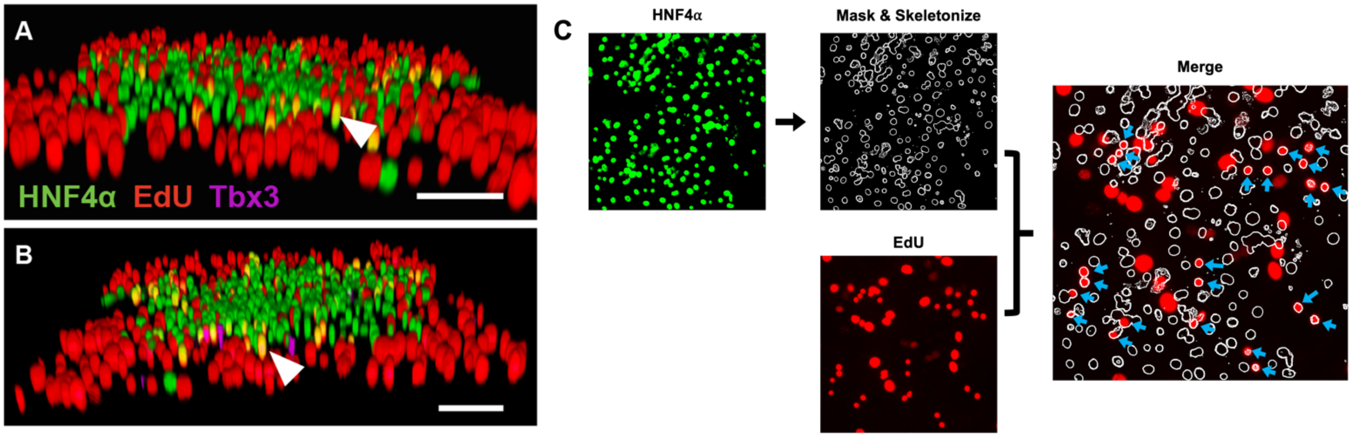

Figure S6

Immunofluorescence analysis of Heps treated with 10 μM PGE_2 indicating (**A**) HNF4α+/EdU+ nuclei (representative overlap is marked with white arrow) and (**B**) HNF4α+/Tbx3+ (representative overlap is marked with white arrow) (confocal 3D rendering, scale bar = 100 μm). (**C**) In order to quantify double positive (HNF4α+/EdU+) nuclei, two different channels were overlaid. The HN4α channel (green) is first masked and skeletonized to outline the nuclei and then overlaid with the EdU channel (red). The double positive nuclei (blue arrows) are then manually scored using an ImageJ counter.

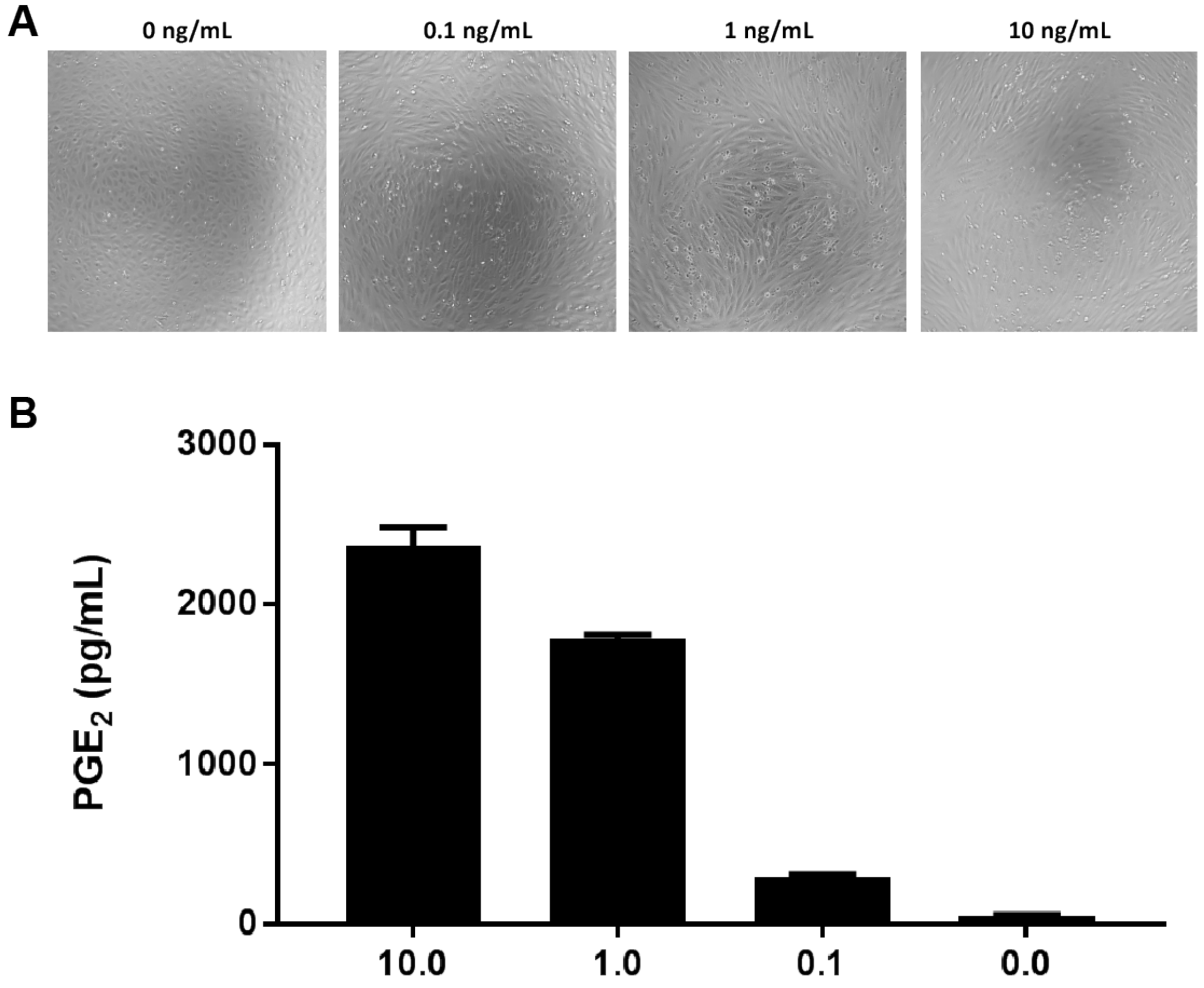

Figure S7

IL1β stimulates HUVECs to produce PGE_2. (**A**) Phase contrast images of HUVECs treated with varying concentrations of IL1β for two days. (**B**) PGE_2 concentrations measured in the supernatant of HUVEC cultures two days after stimulation. Each bar represents a different concentration of IL1β (n = 3 wells, mean ± SEM).

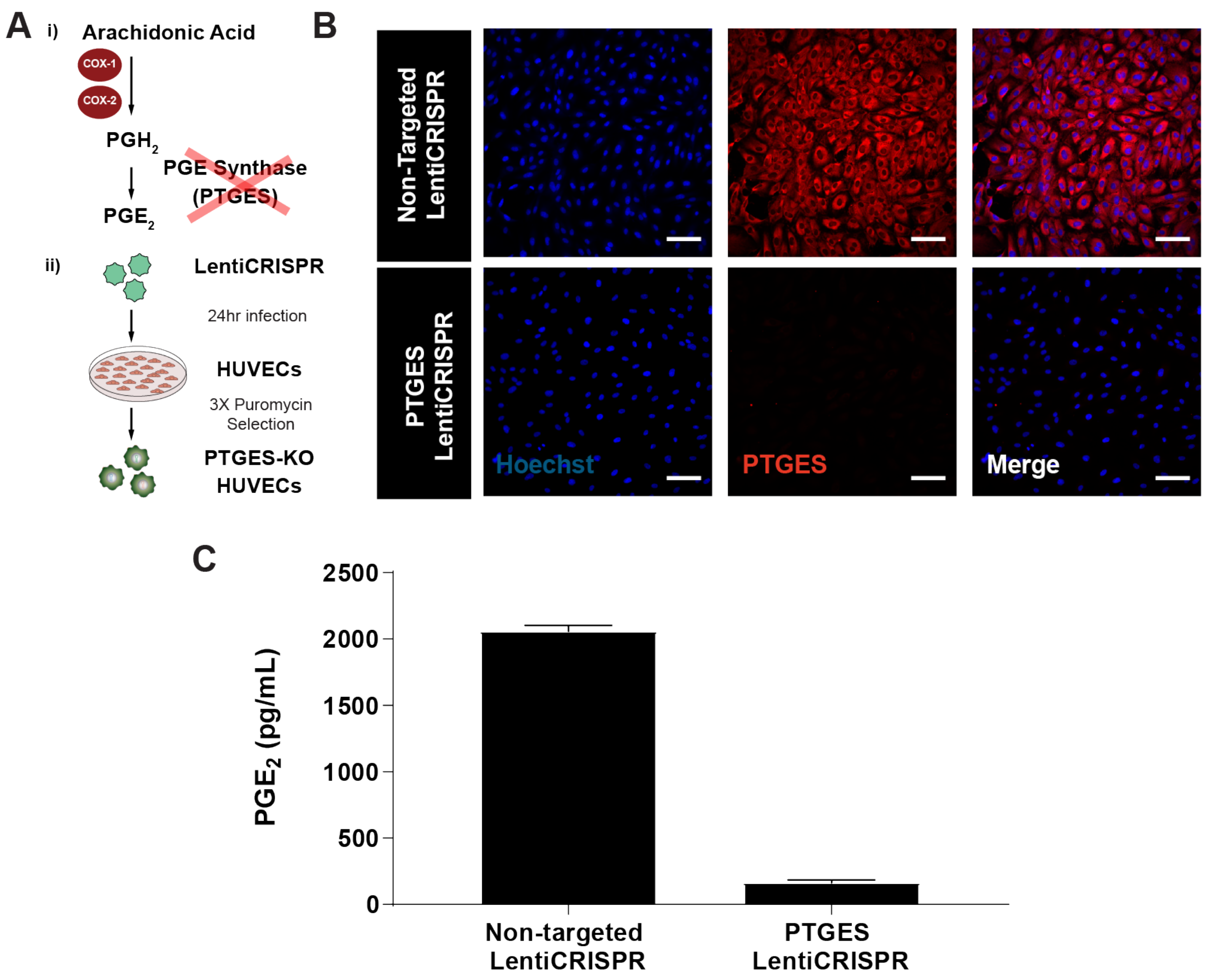

Figure S8

Prostaglandin E Synthase (PTGES) can be reliably knocked out from HUVECs using CRISPR/Cas9. (**A**) (i) Strategy for disrupting PGE_2 biosynthesis in HUVECs. (ii) Lentiviral particles harboring Cas9 and the sgRNA for PTGES are utilized to create a stable knockout line. (**B**) Immunofluorescence analysis of HUVECs targeted by various lentiviral vectors (maximum intensity projections, scale bar = 100 μm). Each HUVEC line has undergone two rounds of puromycin selection. (**C**) PGE_2 concentrations measured in the supernatant of the HUVEC cultures two days after 10 ng/mL IL1β stimulation (n = 3 wells, mean ± SEM).
